# Disrupted gut microbiota aggravates working memory dysfunction induced by high-altitude exposure in mice

**DOI:** 10.3389/fmicb.2022.1054504

**Published:** 2022-11-10

**Authors:** Zhifang Zhao, Dejun Cui, Guosong Wu, Hong Ren, Ximei Zhu, Wenting Xie, Yuming Zhang, Liu Yang, Weiqi Peng, Chunxiao Lai, Yongmei Huang, Hao Li

**Affiliations:** ^1^Department of Gastroenterology, National Institution of Drug Clinical Trial, Guizhou Provincial People's Hospital, Medical College of Guizhou University, Guiyang, Guizhou, China; ^2^Department of Pharmacy, Baiyun Branch, Nanfang Hospital, Southern Medical University, Guangzhou, China; ^3^Plateau Brain Science Research Center, Tibet University, Lhasa, China; ^4^Department of Gastroenterology, Baiyun Branch, Nanfang Hospital, Southern Medical University, Guangzhou, China

**Keywords:** gut microbe, probiotic, high altitude, gut-brain axis, antibiotics

## Abstract

**Background:**

The widely accepted microbiome-gut-brain axis (MGBA) hypothesis may be essential for explaining the impact of high-altitude exposure on the human body, especially brain function. However, studies on this topic are limited, and the underlying mechanism remains unclear. Therefore, this study aimed to determine whether high-altitude-induced working memory dysfunction could be exacerbated with gut microbiota disruption.

**Methods and results:**

C57BL/6 mice were randomly divided into three groups: control, high-altitude exposed (HAE), and high-altitude exposed with antibiotic treatment (HAE-A). The HAE and HAE-A groups were exposed to a low-pressure oxygen chamber (60–65 kPa) simulating the altitude of 3,500–4,000 m for 14 days, The air pressure level for the control group was maintained at 94.5 kPa. Antibiotic water (mixed with 0.2 g/L of ciprofloxacin and 1 g/L of metronidazole) was provided to the HAE-A group. Based on the results of the novel object test and P300 in the oddball behavioral paradigm training test, working memory dysfunction was aggravated by antibiotic treatment. We determined the antioxidant capacity in the prefrontal cortex and found a significant negative influence (*p* < 0.05) of disturbed gut microbiota on the total antioxidant capacity (T-AOC) and malondialdehyde (MDA) content, as well as the activities of superoxide dismutase (SOD) and glutathione peroxidase (GSH-Px). The same trend was also observed in the apoptosis-related functional protein content and mRNA expression levels in the prefrontal cortex, especially the levels of bcl-2, Bax, and caspase-3. The high-altitude environment and antibiotic treatment substantially affected the richness and diversity of the colonic microbiota and reorganized the composition and structure of the microbial community. *S24-7*, *Lachnospiraceae*, and *Lactobacillaceae* were the three microbial taxa with the most pronounced differences under the stimulation by external factors in this study. In addition, correlation analysis between colonic microbiota and cognitive function in mice demonstrated that *Helicobacteraceae* may be closely related to behavioral results.

**Conclusion:**

Disrupted gut microbiota could aggravate working memory dysfunction induced by high-altitude exposure in mice, indicating the existence of a link between high-altitude exposure and MGBA.

## Introduction

With the development of highland areas, numerous people from the plains access the plateau every year for work or tourism. High-altitude environments are characterized by lack of oxygen, low pressure, low temperature, and strong ultraviolet radiation; within these features, the reduction in oxygen content plays an important role in affecting human life activities (Li et al., 2015; Tremblay et al., 2021). High-altitude hypoxia has different effects on the digestive, cardiovascular, and nervous systems, and the metabolism of exogenous substances (Pena et al., 2022). The brain is the body’s largest oxygen-consuming organ, and its cognitive function is significantly adversely affected by lack of oxygen. Studies have shown that high-altitude hypoxic environments can result in changes to various cognitive processes in the brain, such as decreased attention (Svensson et al., 2015; Zhang D. et al., 2018), inhibitory control (Ma et al., 2015), executive function task correctness, and prolonged reaction times. Although high-altitude exposure can impair brain function, effective methods to prevent such an impact in high-altitude areas remain limited.

Medical researchers have developed the concept of the “microbiome-gut-brain axis (MGBA),” which is gaining increasing attention in the basic biological and physiological studies in the field of psychiatry, neurodevelopment, and age-related and neurodegenerative diseases. MGBA is a two-way information exchange system that integrates brain and gut functions, while the central nervous system communicates with the gut microbiota mainly through metabolites of microbial origin (Bravo et al., 2011; Yano et al., 2015). One study found that mice on a high-fat diet exhibited enhanced systemic and central nervous system inflammatory responses, which, in turn, increased the risk of Alzheimer’s disease by affecting the MGBA system, leading to reduced cognitive function and β-amyloid accumulation (Bruce-Keller et al., 2015). In addition, *Bifidobacterium* and *Lactobacillus* have been shown to reduce symptoms associated with anxiety and depression and have a positive effect on memory, learning, and cognition in a variety of animal models (Sun et al., 2015). In addition to the metabolites of the gut microbiota, some of the intermediate products of microbiota metabolism can also cross the blood–brain barrier and act directly on the brain (Haghikia et al., 2016). Thus, modulation of MGBA may be a mechanism for regulating the brain, which provides another potential way to prevent brain dysfunction during high-altitude exposure.

Previous studies have shown that high-altitude environments alter the balance of the intestinal microbiota, causing damage to the intestinal structure and mucosal barrier, ultimately inducing and exacerbating intestinal damage (Zhang W. et al., 2018; Wang et al., 2022). In addition, a study based on 17 healthy men found that the relative abundance of *Prevotella* in the intestines was subject to altitude-dependent changes at 4,300 m, effectively demonstrating that intestinal microbiota may contribute to host variability in response to high-altitude exposure (Ananthakrishnan et al., 2018). The above-mentioned information supports the possibility of considering MGBA as a possible preventive method against brain dysfunction under high-altitude exposure; however, research is needed to demonstrate the role of MGBA during high-altitude exposure.

Therefore, we placed mice in a high-altitude environment simulation device and disrupted the intestinal microbiota of one group of mice using a combination of antibiotics. New object recognition was used to detect working memory impairment and prefrontal oxidative stress markers and apoptotic protein levels were examined. Differences in the composition of intestinal microbiota were analyzed by colonic 16S rDNA sequencing. The present study aimed to provide evidence to answer the following two research questions: (1) Does disturbed gut microbiota exacerbate high-altitude exposure impairment of working memory in mice? (2) The decrease/increase of which gut symbiotic bacteria may play a key role? This study provides a theoretical basis for the application of intestinal commensal bacteria in working memory impairment due to high-altitude exposure.

## Materials and methods

### Animal treatment and sampling

A total of 36 C57BL/6 mice of the same age (8 weeks old, purchased from SPF Biotechnology Co., Ltd., Beijing, China) were randomly selected and divided into three groups after a week of adjustment. The three groups were as follows: control, high-altitude exposed (HAE), and high-altitude exposed with antibiotic treatment (HAE-A). The air pressure level for the control group was maintained at 94.5 kPa, while the HAE and HAE-A groups were exposed to a low-pressure oxygen chamber simulating an altitude of 3,500–4,000 m for 14 days, with the air pressure set to 60–65 kPa. Antibiotic water (mixed with 0.2 g/L of ciprofloxacin and 1 g/L of metronidazole) was provided to the HAE-A group, and the other two groups were provided with normal drinking water during the 14 experimental days. The temperature and humidity of the low-pressure oxygen chamber were identical to those outside the chamber. The chamber was opened for 1 h per day to supply food (Chengdu Dashuo Institute of Biology, Chengdu, China) and water. All animal experiments were conducted in accordance with the guidelines for the feeding and use of experimental animals and approved by the Committee of Nanfang Hospital Baiyun Branch, Southern Medical University (approval no: SYXK2022-023). All animals were kept in a room with a controlled temperature (22 ± 2°C) and a 12/12-h light/dark cycle (dark period: 7 p.m.–7 a.m.).

After the 14 day experimental period, eight mice from each experimental group were randomly selected. After the behavioral test (novel object test, described below), the mice were immediately sacrificed by cervical dislocation, according to the guidelines of the animal care facility. A portion of the prefrontal cortex was immediately removed. These samples were washed in ice-cold sterile saline, frozen in liquid nitrogen, and then stored at −80°C. The rest of prefrontal cortex and the contents of the colon were removed separately and frozen at −20°C until further analysis. Extraction of samples stored at −80°C and prefrontal cortical RNA was performed using the E.Z.N.A. Total RNA Kit (OMEGA Bio-Tek, Doraville, GA, United States) in accordance with the manufacturer’s guidelines. Total RNA (1 μg) was synthesized into first-strand complementary DNA (cDNA) using the PrimeScript™ RT reagent kit with gDNA Eraser (TaKaRa, Dalian, Liaoning, China). The cDNA products were stored at −20°C until subsequent tests.

### Novel object test

**Figure 1:**
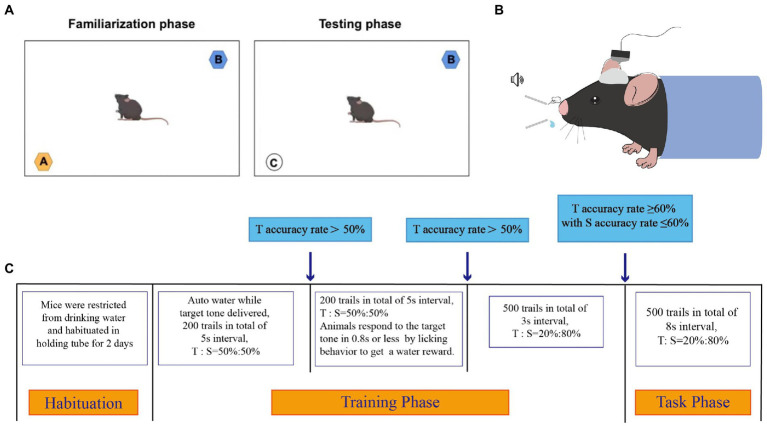
The schematic diagram of novel object test and oddball paradigm behavior training. **(A)** Schematic diagram of novel object test. A dark open-field box (40 * 40 * 45 cm) was used. The objects shown to the mice were a smooth pebble, a blue cap of the same size and form, and a orange cap of the same size and form, respectively. **(B)** Schematic diagram of oddball paradigm behavior training. **(C)** Schematic presentation of different phases of the Oddball paradigm behavior Training. Animals had to correctly respond to infrequent target (8 Hz) tones while correctly ignoring infrequent standard (6 Hz) tones. The electrical signals of the mice were recorded during the task phase. T referred to Target tone, S referred to Standard tone.

The schematic diagram of novel object test is shown in Figure 1A. Mice tend to investigate novel objects rather than familiar ones. On this basis, a novel object test of working memory formation was conducted using the method described by Gareau et al. (2011), with minor modifications. Briefly, the mice were placed in a dark open-field arena (40 × 40 × 45 cm, length × width × height) and allowed to explore freely for 1 h for habituation. The mice were shown two distinct objects: a smooth pebble of the right size and blue and orange tube caps of the same size and form. The phases of behavioral assessment included familiarization and testing.

During familiarization, the blue and orange tube caps were placed in the opposing corners of the arena, and the mice were permitted to examine both items for 5 min. After the objects had been removed, the mice were given a 20-min rest before testing.

The orange tube cap was replaced with a smooth pebble during the rest period and during testing, the mice were exposed to the blue tube cap and the smooth pebble. Memory can be assessed as the frequency with which the smooth pebble is explored in comparison with the blue tube cap. The exploration ratio is a measure of how often mice sniff new objects compared to old ones (ratio of the frequency of sniffing the smooth pebble to the total frequency of sniffing the blue tube cap and smooth pebble). No difference between the two objects, or a ratio of 0.5, indicated impaired hippocampus-dependent memory. In the present test, exploration was defined as orientation toward the object with the nose pointing directly at the object within 1–2 cm.

### Biochemical analysis

The antioxidant indices in the prefrontal cortex were measured using commercial kits (Jiancheng Bioengineering Institute, Nanjing, Jiangsu, China), including the activities of catalase (CAT), total antioxidant capacity (T-AOC), glutathione peroxidase (GSH-Px), superoxide dismutase (SOD), malondialdehyde (MDA), and GSH content (Zareie et al., 2006). The levels of bcl-2 and Bax in the prefrontal cortex were determined by ELISA using murine-specific reagent kits (MLBIO Biotechnology Co., Ltd., Shanghai, China). Data were analyzed using individual mice. Statistical analyses were performed using one-way ANOVA, followed by Duncan’s multiple-range test for multiple comparisons (satisfying both normality and equal variance tests; SigmaPlot for Social Sciences version 12). Differences were considered statistically significant at *p* < 0.05.

### Real-time quantitative polymerase chain reaction analysis

The cDNA products generated from the prefrontal cortex were determined by PCR. A CFX96 Real-time PCR Detection System (Bio-Rad, Hercules, CA, United States) with iTaq Universal SYBR Green Supermix (Bio-Rad, Hercules, CA, United States) was used with the following protocol: 5 min at 95°C, 40 cycles of 10 s denaturation at 95°C, and 30 s annealing/extension at optimum temperature (Table 1). The PCR product purity was analyzed using a final melting curve analysis. Standard curves were generated using serial dilutions. Primer sequences for the target genes are shown in Table 1. The mRNA abundance was estimated using the _ΔΔ_Ct method. The samples (*n* = 6) in each group were analyzed in triplicate, and Ct was calculated as (Ct_target_ − Ct_β-actin_)_treatment_ − (Ct_target_ − Ct_β-actin_)_control._ β-actin was used as a eukaryotic housekeeping gene to normalize relative gene expression levels. The measured mean values were used to assess mRNA expression levels of Bcl-2, Bad, Bax, caspase-3, and caspase-9 in the prefrontal cortex. Data were analyzed using individual mice. Statistical analyses were performed using one-way analysis of variance (ANOVA), followed by Duncan’s multiple-range test for multiple comparisons (satisfying both normality and equal variance tests; SigmaPlot for Social Sciences version 12). Differences were considered statistically significant at *p* < 0.05.

**Table 1:** Primer sequences for RT-qPCR in the prefrontal cortex.

Gene	Tm (°C)	Sequence
β-actin	60	F: GCTCTTTTCCAGCCTTCCTT
		R: GATGTCAACGTCACACTT
caspase-9	61	F: GAGGTGAAGAACGACCTGAC
		R: AGAGGATGACCACCACAAAG
caspase-3	59	F: ACATGGGAGCAAGTCAGTGG
		R: CGTCCACATCCGTACCAGAG
Bax	61	F: ATGCGTCCACCAAGAAGC
		R: CAGTTGAAGTTGCCATCAGC
Bad	60	F: AGAGTATGTTCCAGATCCCAG
		R: GTCCTCGAAAAGGGCTAAGC
bcl-2	61	F: AGCCTGAGAGCAACCCAAT
		R: AGCGACGAGAGAAGTCATCC

### P300 in the oddball behavioral paradigm training test

#### Surgery and treatment

Before high-altitude exposure, another six mice per group (single-caged) were anesthetized with isoflurane (O.D, 1%–3% in oxygen, R580 anesthesia machine, RWD) and placed on a stereotaxic frame (680303, RWD). A water circulation insulation system (68662, RWD) was used to maintain body temperature at 37°C throughout the experiment. Ophthalmic gel was used to protect the eyes from drying. The heads of the mice were shaved at the surgical area, which was cleaned using 75% ethanol. The skull was exposed horizontal to the bregma and lambda landmarks. Bregma measurement was performed to determine the anterior–posterior (AP) and medial–lateral (ML) coordinates for electrode implantation. Four screws were secured to the skull, one to serve as the recording ground, and the other three to fix the dental cement. A 16-channel Microwire Electrode Array (MEA, arranged in the 2 × 8 configuration, 33 μm diameter nickel-chromium wires with formvar insulation, 0.25 mm inter-electrode spacing, KedouBC), was implanted into the PFC (A/P + 1.0 mm L/M + 0.4 mm D/V −1.8 mm), and secured with dental cement to a home-made metal plate to immobilize the head for oddball behavioral paradigm training. Mice in the HAE and HAE-A groups experienced high-altitude exposure 7 days after recovery from surgery.

#### Oddball paradigm behavior training

After full recovery from surgery, head-fixed mice were trained in a hearing-based bionic stimulation oddball paradigm. A pair of sound stimuli (~86 db) was used: the target tone (8 kHz) and standard tone (6 kHz). All the tone stimulations were marked using a microcontroller. The target tone was set to be associated with the water reward, and the standard tone was set to be associated with air puff punishment. In the target trial, the water valve was switched on for 500 ms immediately after the detection of the animal’s licking behaviors within the 800 ms response window, and the mouse was rewarded with 10–15 μl water. In the standard trial, the animal’s licking behavior triggered an air puff of 500 ms. The training protocol and recording process are shown in Figures 1B,C.

### Data acquisition and analysis

Electrophysiological signals were recorded using the Plexon Data Acquisition System (Plexon, United States). Signals were amplified (1,000 Ã–) and bandpass filtered (0.5–30 Hz) and acquired at 2 k Hz for off-line analysis. The LFP responses to the target and standard stimulation were averaged to obtain event-related potentials (ERP). Stimulus-locked trials were extracted for all instances, with each trial comprising up to 800 ms of data and a −200 ms prestimulus. A semi-automated algorithm was used to select discernable ERP peaks, which were baseline-corrected using a 200 ms prestimulus interval. Considering the effect of artifacts on the P3 amplitude/latency estimate, the average of 10 ms before and after the peak in the time window of 250–400 ms was defined as the amplitude of P300, and the time point of the peak was defined as the delay of P300. The peak amplitudes and latencies of P300-like components were calculated for each mouse. Data are presented as mean ± standard deviation (SD). Data between the two groups were compared using paired or unpaired *t*-tests, as appropriate. Repeated-measures (RM) analysis of variance (ANOVA) was used for statistical analyses of the normalized data. Statistical significance was set at *p* < 0.05. If a significant main or interaction effect was found, Newman–Keuls *post-hoc* test was applied.

### Bioinformatic analysis of gut microbiota

Total DNA samples of colon contents were obtained using the E. Z. N. A. TM fecal DNA extraction kit (OMEGA Bio-Tek, Norcross, GA, United States), and the final elution volume was 100 μl. The integrity and quality of extracted DNAs were measured using NanoDrop NC2000 spectrophotometer (Thermo Fisher Scientific, Waltham, MA, United States) and agarose gel electrophoresis, respectively. DNA samples were sent to Shanghai Personal Biotechnology Co., Ltd. (Shanghai, China). The specific barcode was incorporated into the amplification primers, and then the bacterial 16S rRNA gene was amplified by PCR and detected by pair-end 2 × 250 bp using the Illumina MiSeq sequencing platform and V3-V4 hypervariable region fragment. The primer sequences used were 338F (5′-ACTCCTACGGGGAGGAGCA-3′) and 806R (5′-GGACTachVGGGTWTCTAAT-3′). PCR amplification system: Fast Pfu DNA Polymerase (5 U/μl) 0.25 μl, dNTPs (2.5 mM) 2 μl, buffer (5×) 5 μl, DNA template 1 μl, ddH2O 14.75 μl forward and reverse primers (10 μM) 1 μl. Amplification program settings consisted of initial denaturation at 98°C for 5 min, 25 cycles; denaturation at 98°C 30 s; annealing at 53°C 30 s; extension 72°C 45 s; and extension at 72°C 5 s. The Quant-iT PicoGreen dsDNA Assay Kit (Invitrogen, Carlsbad, CA, United States) and Vazyme VAHTSTM DNA Clean Beads (Vazyme, Nanjing, China) were used to quantify and purify the PCR amplicons, respectively.

The tool QIIME2 (Bolyen et al., 2018) was used to perform bioinformatic analysis of raw data in reference to the official tutorial.^1^ The feature-classifier plugin (Price et al., 2009) based on the Navier Bayes classifier and Greengenes13_8 database was used to annotate the species in the abundance table. Downstream analysis was conducted primarily using Rstudio software (V3.1.2). The alpha diversity index (observed species number and Shannon diversity) was calculated with ASVs abundance, using the vegan package. For in-depth analyses, we filtered sequences with at least eight sequences (number of replicates per treatment) of ASVs counts and relative abundance exceeding 0.1% ASV in all samples as further data for standardized pretreatment. The normalized step for filtered ASV sequence counts was performed using the TMM method in the edgeR package (Robinson et al., 2010). The structure of the gut community was assessed by principal coordinate analysis (PCoA) based on Bray–Curtis dissimilarity using the phyloseq package (McMurdie and Holmes, 2013). Furthermore, permutational multivariate analysis of variance (PERMANOVA) was applied to evaluate the differences in intestinal microbial communities among different treatments using the vegan package with the adonis method. Microbial taxonomy at the phylum and family levels was clustered among the different treatments. In addition, we used indicator species analysis to obtain the point-biserial correlation coefficient (r) of an ASV positively associated with one or more treatments. *p* < 0.05 was set as the filtering threshold to identify the key species, and the bipartite network diagram was visualized. To observe the biomarkers of gut communities in the highland environment and antibiotic treatment, we applied LEfSe analysis to detect differentially abundant taxa across groups (LDA > 4.0, *p* < 0.05). Finally, the Spearman’s correlation coefficient (*r* > 0.7 and *p* < 0.05) and Mantel test were calculated to determine the association between the microbial community and behavioral results for all treatments.

## Results

### Behavioral test and P300 amplitude

**Figure 2:**
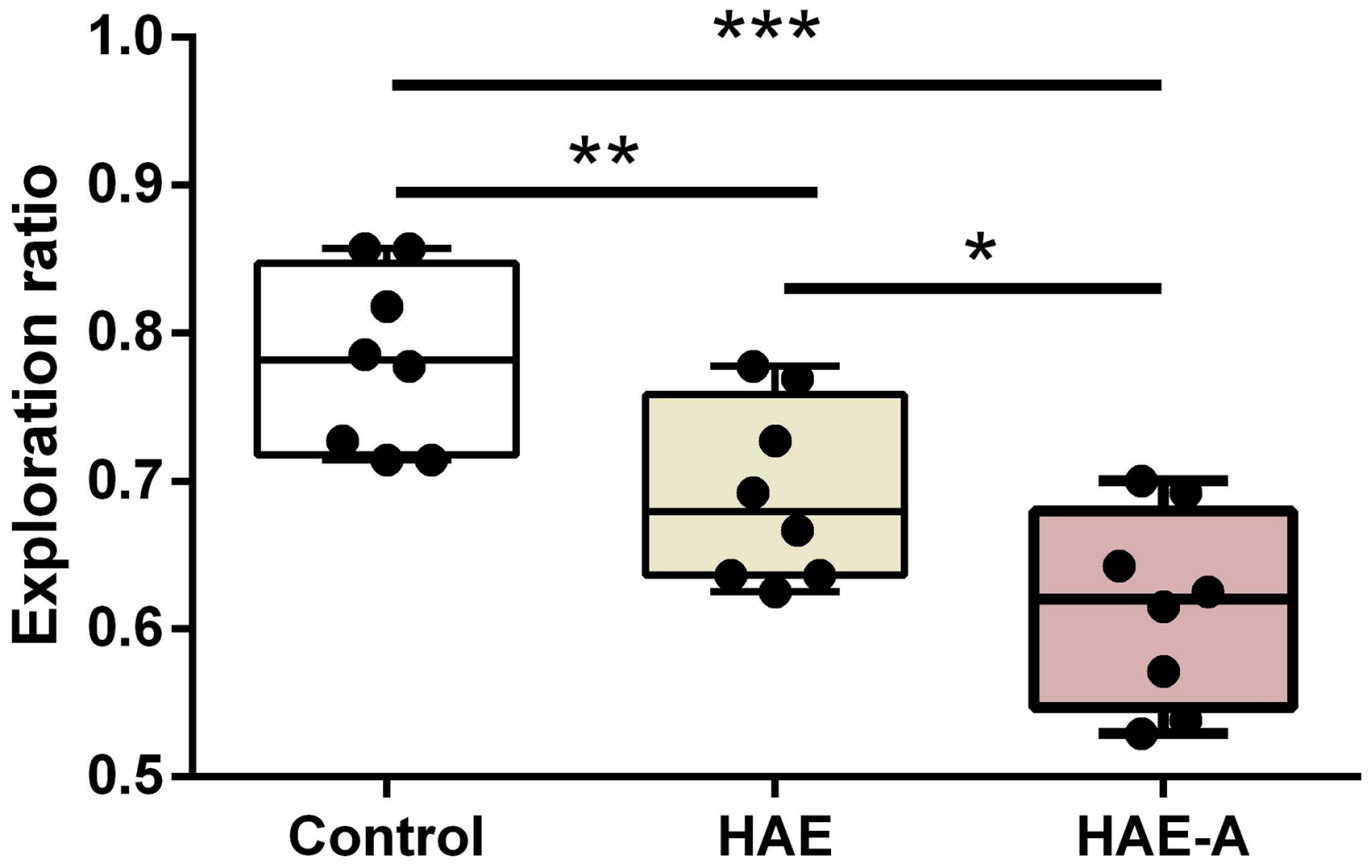
Effects of high altitude and antibiotics on exploration ratio by novel object test. Data are presented with the means ± standard deviation (*n* = 8). *Difference is significant at the 0.05 level (*p* < 0.05); **difference is significant at the 0.01 level (*p* < 0.01); ***difference is significant at the 0.001 level (*p* < 0.001).

**Figure 3:**
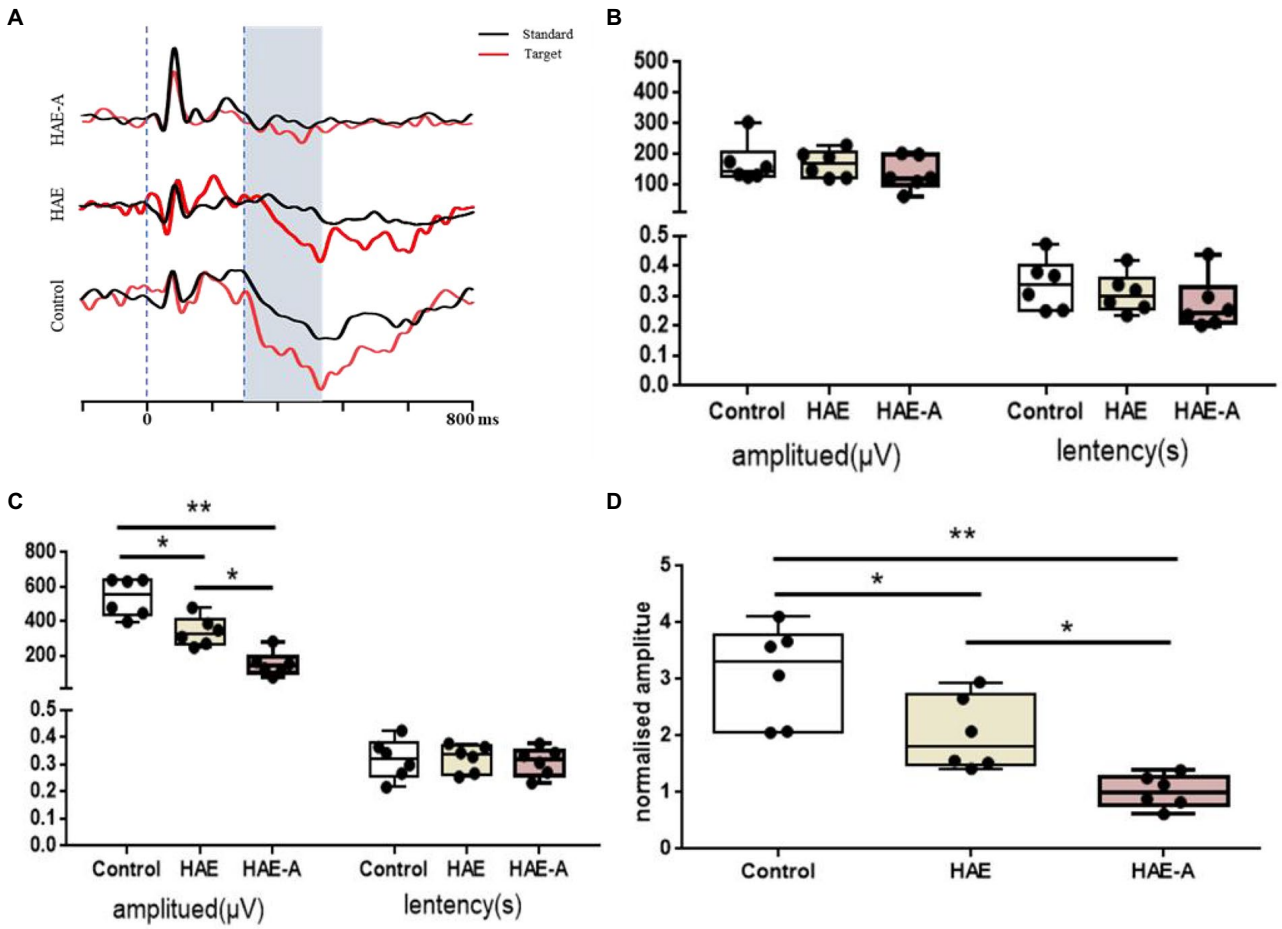
Variation of P300 of the oddball behavioral paradigm training test. **(A)** The profile of P300 in control, HAE and HAE-A group, respectively; **(B)** The result of P300 evoked by standard tone stimulation; **(C)** The result of P300 evoked by target tone stimulation; **(D)** The result of normalized P300 amplitude by target/standard. Data are presented with the means ± standard deviation (*n* = 6). *Difference is significant at the 0.05 level (*p* < 0.05); **difference is significant at the 0.01 level (*p* < 0.01).

Figure 2 shows the results of the behavioral tests for memory capacity in mice. The exploration rate in the NOR trials was significantly lower in the HAE (*p* < 0.01) and HAE-A groups (*p* < 0.001) than that in the control group. In addition, the exploration rate was significantly lower in the HAE-A group than that in the HAE group (*p* < 0.05). Moreover, we collected the P300 event-related potential (ERP) component during performance of the oddball paradigm behavior training task. After oddball paradigm behavior training, control, HAE, and HAE-A mice exhibited a clear P300 peak amplitude during both standard and target tones. As expected, both amplitude (*n* = 6, *p* > 0.005, control: 170.24 ± 67.91, HAE: 167.04 ± 44.95, HAE-A: 135.64 ± 54.62) and latency (*n* = 6, *p* > 0.005, control: 0.34 ± 0.09, HAE: 0.31 ± 0.07, HAE-A: 0.27 ± 0.09) of standard tone were not significantly different among the three groups. As for target tone, the amplitudes were higher than those of standard tone in all groups. The control group showed the highest P300 peak amplitude (539.30 ± 110.40), while the mean P300 peak amplitude (150.62 ± 71.62) of the HAE-A group was the lowest among the three groups and the average P300 peak amplitude of HAE group was 339.88 ± 85.04. The P300 amplitude of both HAE and HAE-A mice was significantly lower than that of the control mice (HAE vs. control: *p* < 0.005, HAE-A vs. control: *p* < 0.001). In addition, the P300 amplitude in HAE mice was significantly higher than that in HAE-A mice (*p* < 0.005). There was no significant difference in the P300 latency of the target tone between the three groups (*n* = 6, *p* > 0.005, control: 0.32 ± 0.07, HAE: 0.32 ± 0.05, HAE-A: 0.3 ± 0.05). In summary, the changes in normalized amplitude (target amplitude/standard amplitude, control: 3.09 ± 0.86, HAE: 2.03 ± 0.65, HAE-A: 1.02 ± 0.29) in all groups were similar to the target amplitude. Compared to control mice, the normalized amplitudes of HAE and HAE-A were significantly decreased (HAE vs. control: *p* < 0.005, HAE-A vs. control: *p* < 0.001). Furthermore, the normalized amplitude of HAE-A was significantly lower than that of HAE (*n* = 6, *p* < 0.005). The amplitude and latency of the P300 in each group are shown in Figure 3.

### Antioxidant capacity in the prefrontal cortex

**Figure 4:**
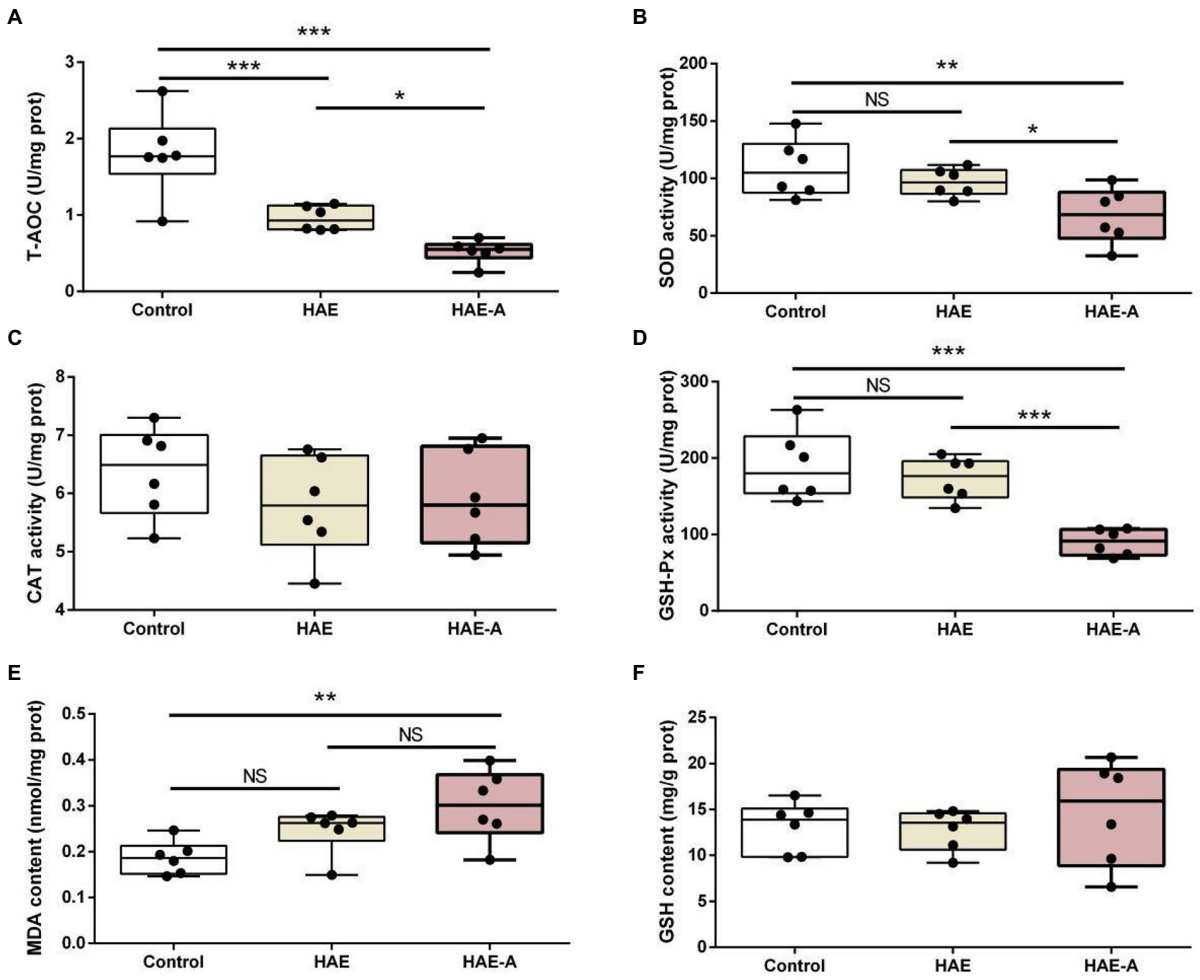
Antioxidant capacity in the prefrontal cortex. Data are presented with the means ± standard deviation (*n* = 6). NS, not significant (*p* > 0.05); *difference is significant at the 0.05 level (*p* < 0.05); **difference is significant at the 0.01 level (*p* < 0.01); ***difference is significant at the 0.001 level (*p* < 0.001). **(A–F)** Activities or contents of T-AOC, SOD, CAT, GSH-Px, MDA, and GSH, respectively. T-AOC, total antioxidation capacity; SOD, superoxide dismutase; CAT, catalase; GSH-Px, glutathione peroxidase; MDA, malondialdehyde; GSH, glutathione.

Figure 4 shows the antioxidant indices in the prefrontal cortex. As shown in Figure 4A, the T-AOC content was significantly lower in the HAE and HAE-A groups than in the control group (*p* < 0.001), whereas the T-AOC content was significantly lower (*p* < 0.05) in the HAE-A group than in the HAE group. Figures 4B,D show that there was no significant difference in SOD activity and GSH-Px activity between the control and HAE groups (*p* > 0.05), but the SOD activity and GSH-Px activity were significantly lower in the control group (*p* < 0.01 and *p* < 0.001, respectively) and HAE group (*p* < 0.05 and *p* < 0.001, respectively) than in the HAE-A group. Figure 4E shows that there was no significant difference (*p* > 0.05) in MDA content between the control and HAE and HAE and HAE-A, but the MDA content was significantly lower in the control (*p* < 0.01) than that in the HAE-A group. In addition, as shown in Figures 4C,F, CAT activity and GSH content were not significantly different between the three groups (*p* > 0.05).

### Apoptosis-related proteins in the prefrontal cortex

**Figure 5:**
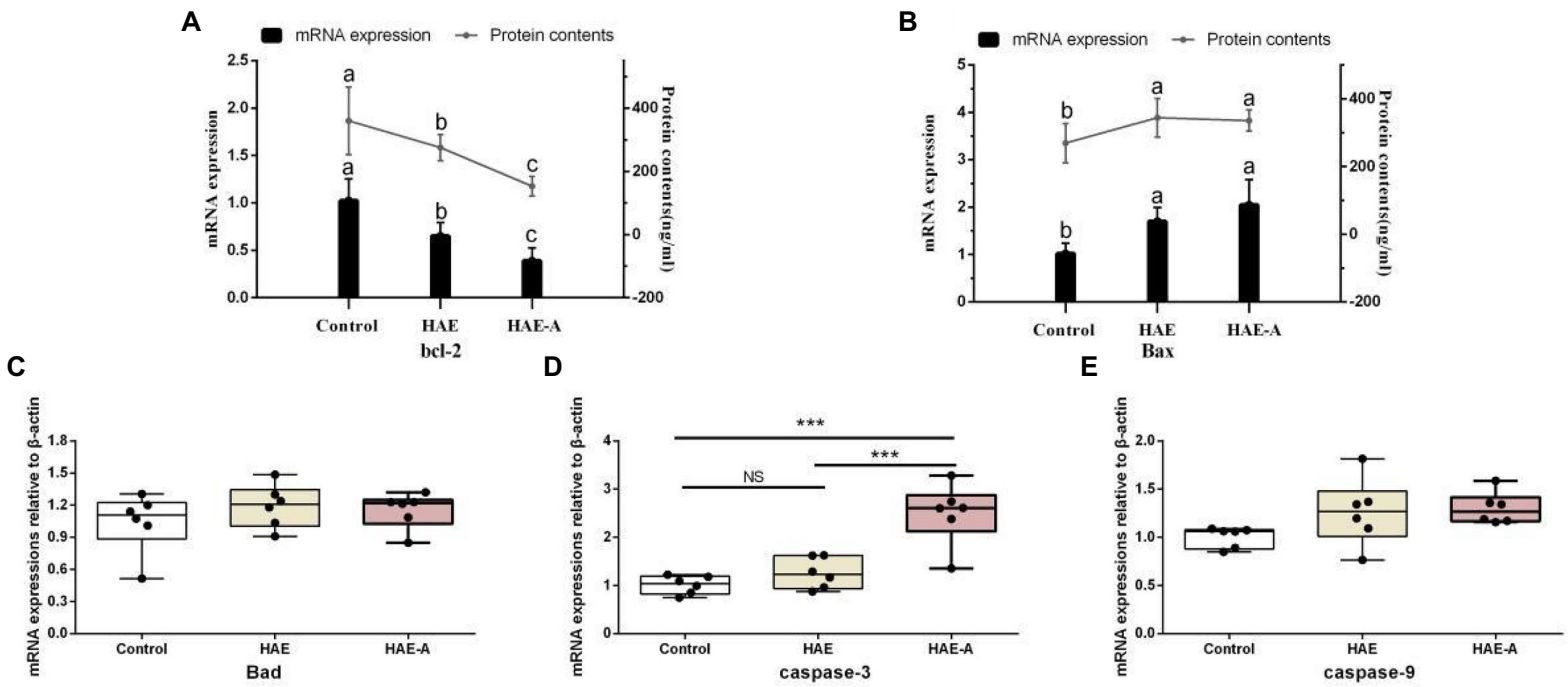
Apoptosis-related functional protein contents and mRNA expression levels in the prefrontal cortex. Data are presented with the means ± standard deviation (*n* = 6). Bars with different letters are significantly different on the basis of Duncan’s multiple-range test (*p* < 0.05). NS, not significant (*p* > 0.05); *difference is significant at the 0.05 level (*p* < 0.05); **difference is significant at the 0.01 level (*p* < 0.01); ***difference is significant at the 0.001 level (*p* < 0.001). **(A,B)**: mRNA expression levels and protein contents of bcl-2 and Bax; **(C–E)**: mRNA expression levels of Bad, caspase-9 and caspase-3, respectively.

Figure 5 shows the apoptosis-related functional protein content and mRNA expression levels in the prefrontal cortex. Among them, the bcl-2 content and bcl-2mRNA expression levels in the HAE and HAE-A groups were significantly lower (*p* < 0.05) than those in the control group, whereas the bcl-2 content and bcl-2mRNA expression levels in the HAE group were significantly higher (*p* < 0.05) than those in the HAE-A group (Figure 5A). Bax and Bax mRNA expression levels were not significantly different (*p* > 0.05) between the HAE and HAE-A groups (Figure 5B). As shown in Figure 5D, the mRNA expression levels of caspase-3 were lower in the control and HAE groups those in the HAE-A group (*p* < 0.001), whereas this indicator did not differ between the control and HAE group (*p* > 0.05). Furthermore, the mRNA expression levels of Bax (Figure 5C) and caspase-9 (Figure 5E) were not significantly different among the three groups (*p* > 0.05).

### Differences in the gut community in microbial compositions and structure

**Figure 6:**
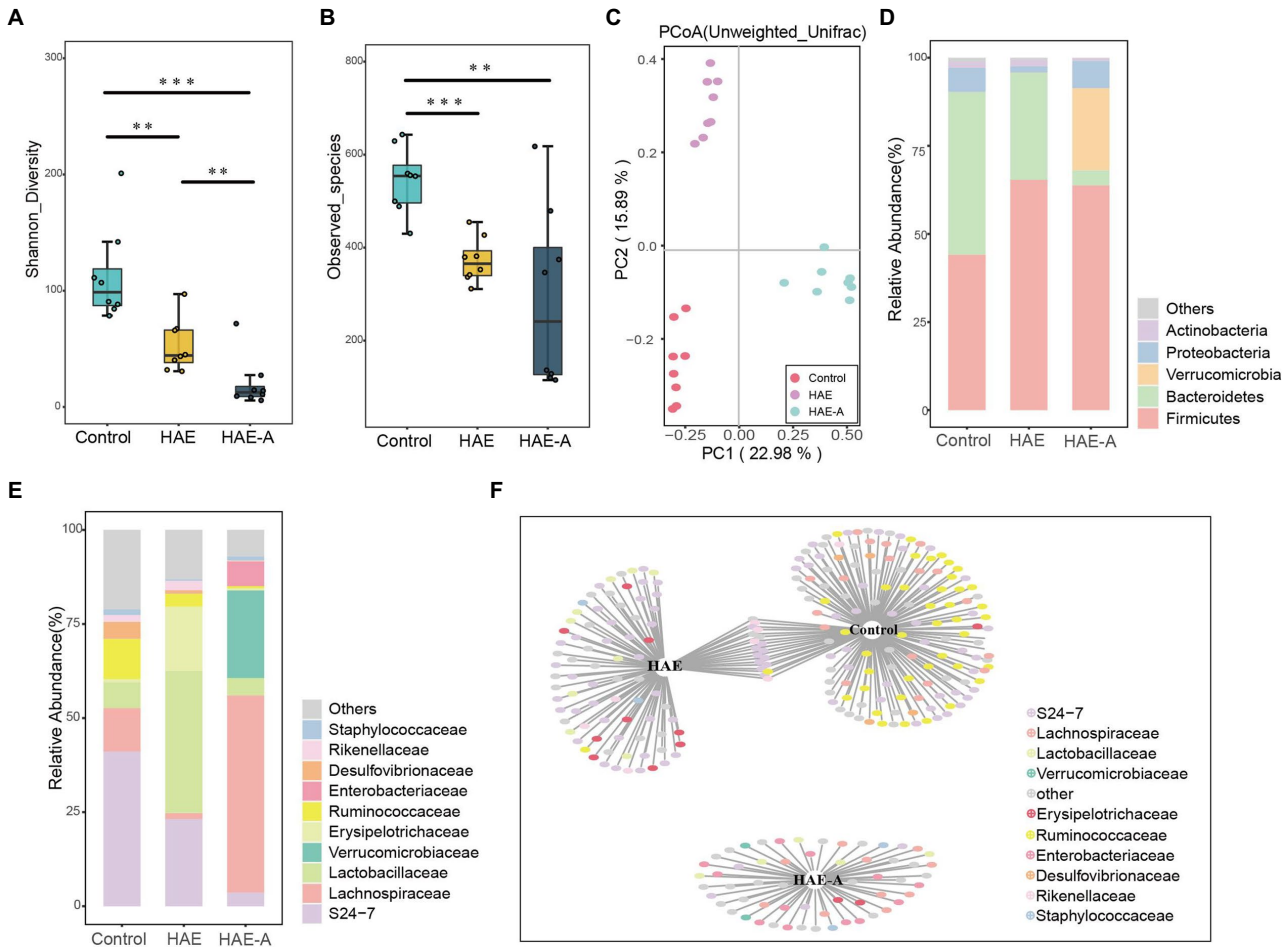
Effects of hypoxia on the structure of colon microbiota in normal mice and antibiotic drinking mice. **(A)** Intestinal microbial community diversity in each group (Shannon). Meaning: Wilcoxon. **(B)** Gut microbiome richness (observed species) in colonic luminal samples of each group. Significance: Wilcoxon. **(C)** Principal Coordinate Analysis (PCoA) of Unweighted UniFrac Distance between groups. **(D)** Relative abundance at phylum level in each group (%). **(E)** Relative abundance at each family level (%). **(F)** Bipartite networks of indicator species analysis. It displays different treatment-specific ASVs in the colonic bacterial communities using indicator species analysis. Circles represent individual bacteria and ASVs that are positively and significantly associated (*p* < 0.05) with one or more different grouping factors (association(s) given by connecting lines). ASVs are colored according to their Family assignment.

There were significant differences in the Shannon diversity index of the colon among the three groups (*p* < 0.01 or *p* < 0.001; Figure 6A). With respect to richness (observed_species; Figure 6B), the control group was significantly different from the other two groups (*p* < 0.01 and *p* < 0.001). We tested the separation pattern (β-diversity) between microbial communities using unconstrained principal coordinate analysis (PCoA) of the Bray–Curtis distance (Figure 6C). The colonic microbial communities of the three groups showed clear separation along the first (22.98%) and second (15.89%) principal coordinates. The findings also showed that the treatment methods of the three groups resulted in completely different microbiota structures (Table 2). In the first principal coordinate, the HAE-A group showed significant separation compared to the other two groups, suggesting that antibiotics were the largest source of variation in the colon microbiome of mice. In addition, the second principal coordinate showed a significant separation in all three groups. The results showed that the combined action of plateau and antibiotics was the second major source of variation in distinguishing the different treatments. In the classification analysis of the colon, there were also significant differences in the intestinal microbiota between the control, HAE, and HAE-A groups. Five bacterial phyla (Figure 6D) were identified as the dominant species in colon samples: *Firmicutes*, *Bacteroidetes*, *Verrucomicrobia*, *Proteobacteria*, and *Actinobacteria*. *Firmicutes* were the dominant species, and *Bacteroidetes* was the second most dominant species. In addition, *Verrucomicrobia* was the third most dominant species. At the family level (Figure 6E), *S24-7*, *Lachnospiraceae*, *Lactobacillaceae*, *Verrucomicrobiaceae*, *Erysipelotrichaceae*, *Ruminococcaceae*, *Enterobacteriaceae*, *Desulfovibrionaceae*, *Rikenellaceae*, and *Staphylococcaceae* were the dominant species.

**Table 2:** PCoA diversity difference test.

Group1	Group2	Sample size	Permutations	pseudo-*F*	Value of *p*	*q*-value
Control	HAE	16	999	5.294379	0.001	0.001
Control	HAE-A	16	999	7.477914	0.002	0.001
HAE	HAE-A	16	999	6.050981	0.001	0.001

**Figure 7:**
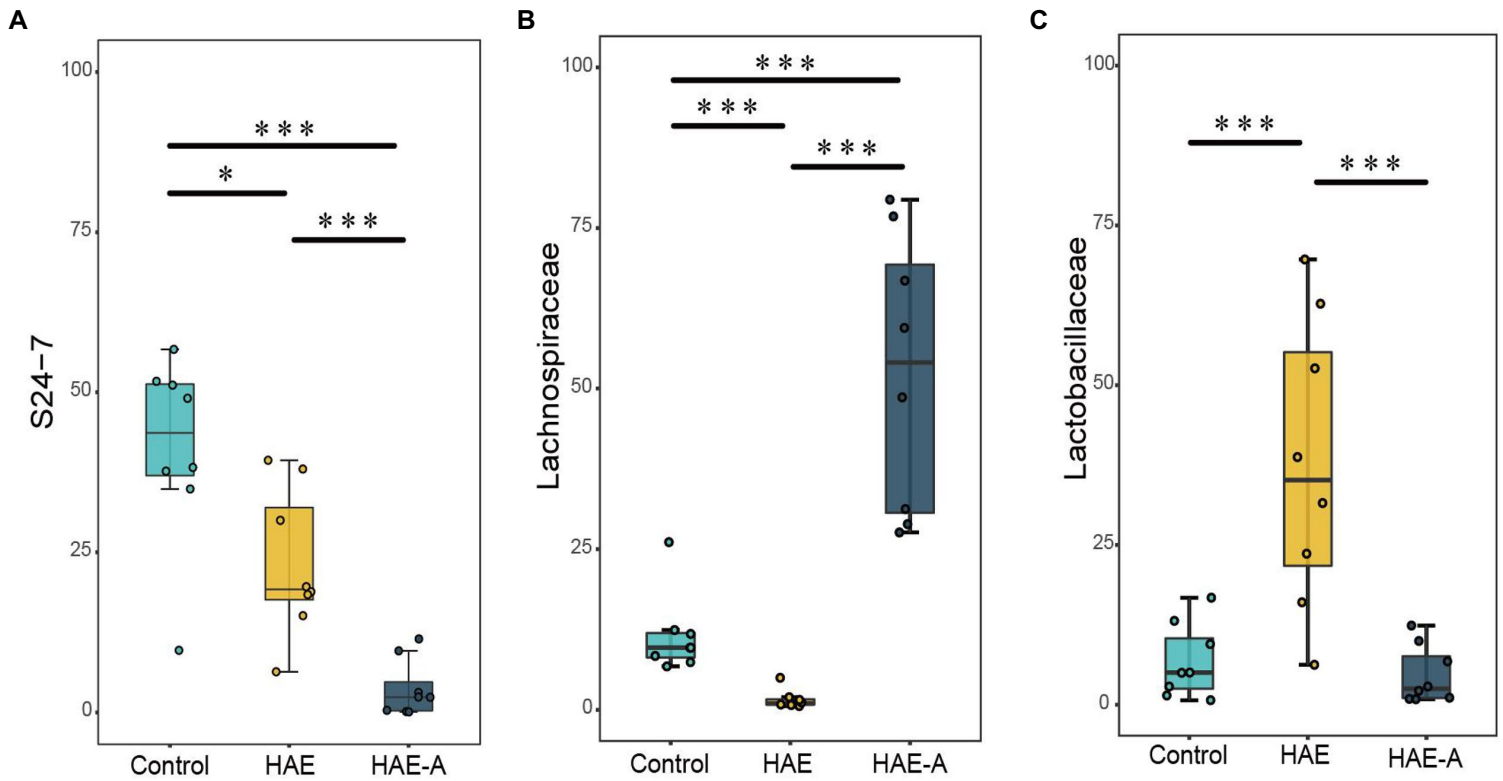
Significant altered bacterial taxa in colonic microbes by different treatments at family level. **A–C**: *S24-7, Lachnospiraceae*, and *Lactobacillaceae*, respectively. Boxplots showing differences in the relative abundance of significantly discriminant taxa between different treatments. Significance between groups was indicated with asterisk (*p < 0.05, **p < 0.01, ***p < 0.001).

**Figure 8:**
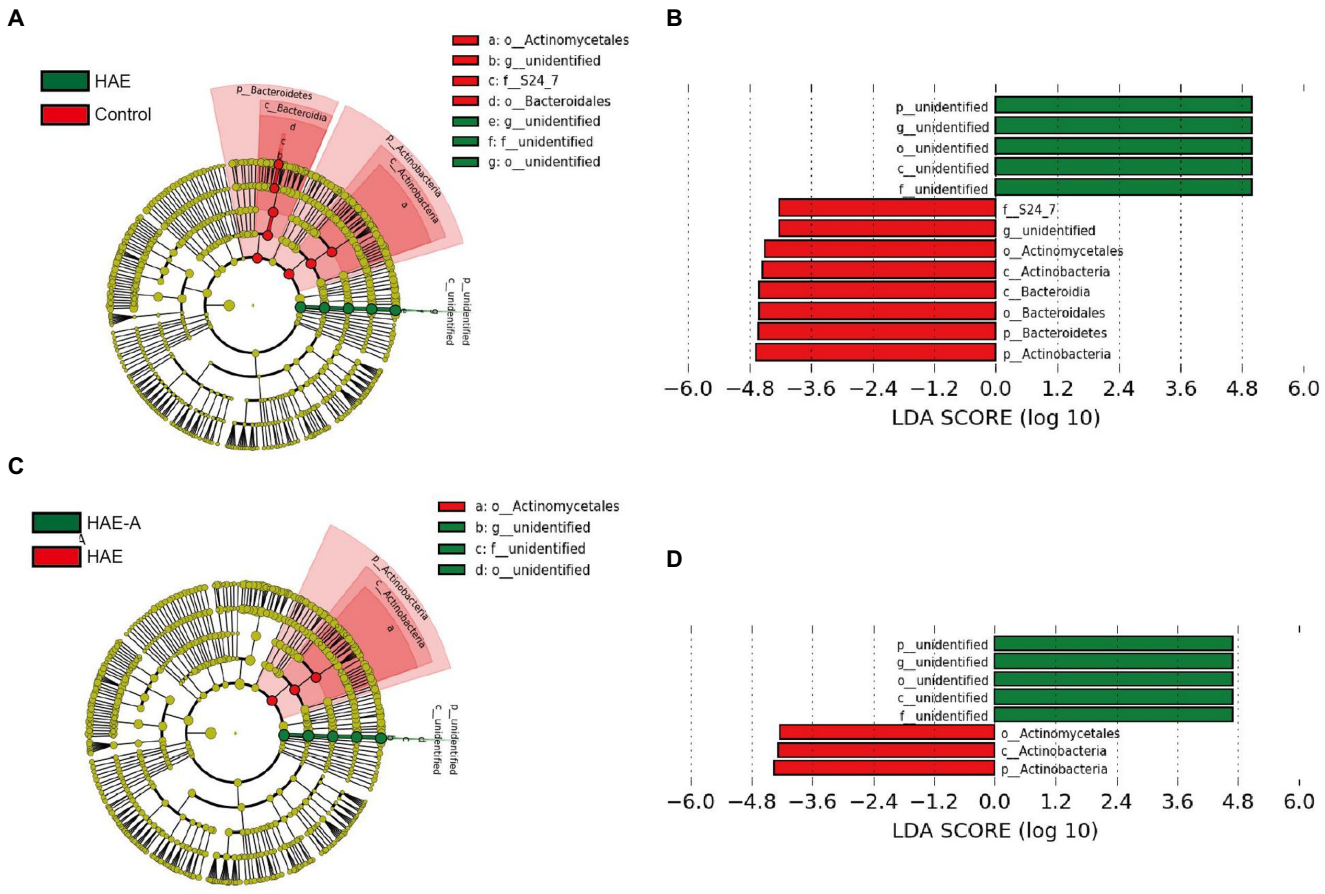
LEfSe analysis of colon microbiota in mice. Cladogram **(A,C)** and linear discriminant analysis (LDA) score **(B,D)** and cladogram **(A,C)** were generated from LDA effect size. Taxa with LDA values larger than 4 are shown in the figure.

*S24-7* was the first dominant species, *Lachnospiraceae* was the second dominant species, and *Lactobacillaceae* was the third dominant species (Figure 6E). Indicator species analysis was also performed to select individual bacteria (ASVs) in the colon microbiota with significant differences in abundance under different treatments, and to perform visual analysis with a bipartite network (Figure 6F). The high number of colonic bacterial ASVs shared between the control and HAE groups reflects the similar clustering of the two sample types in the bacterial community, but bacterial ASVs shared by HAE and control samples, as well as HAE and HAE-A samples, were completely absent. Family-level cluster analysis was conducted for indicator species, and *S24-7*, *Lachnospiraceae*, and *Lactobacillaceae* belonging to indicator species were selected in the colon segment (Figures 6F, 7). Among them, *Lactobacillaceae* was most closely related to the damage caused by the high-altitude environment, and *Lachnospiraceae* was most closely related to the damage caused by antibiotic use in the plateau environment, compared with the control group. *S24-7* was the most deficient bacterium in the HAE and HAE-A groups. LEfSe is an algorithm for the discovery of biomarkers that identify genomic features (genes, pathways, or taxa) that characterize the differences between two or more biological conditions. Compared with the HAE group (Figures 8A,B), the significant discriminative taxa in the control group were *Actinomycetales*, *S24-7*, *and Bacteroidales*. Compared with the HAE-A group (Figures 8C,D), the significant discriminative taxa were *Actinomycetales* and *Actinobacteria* in the HAE group.

### Correlation between gut community and behavioral test

**Figure 9:**
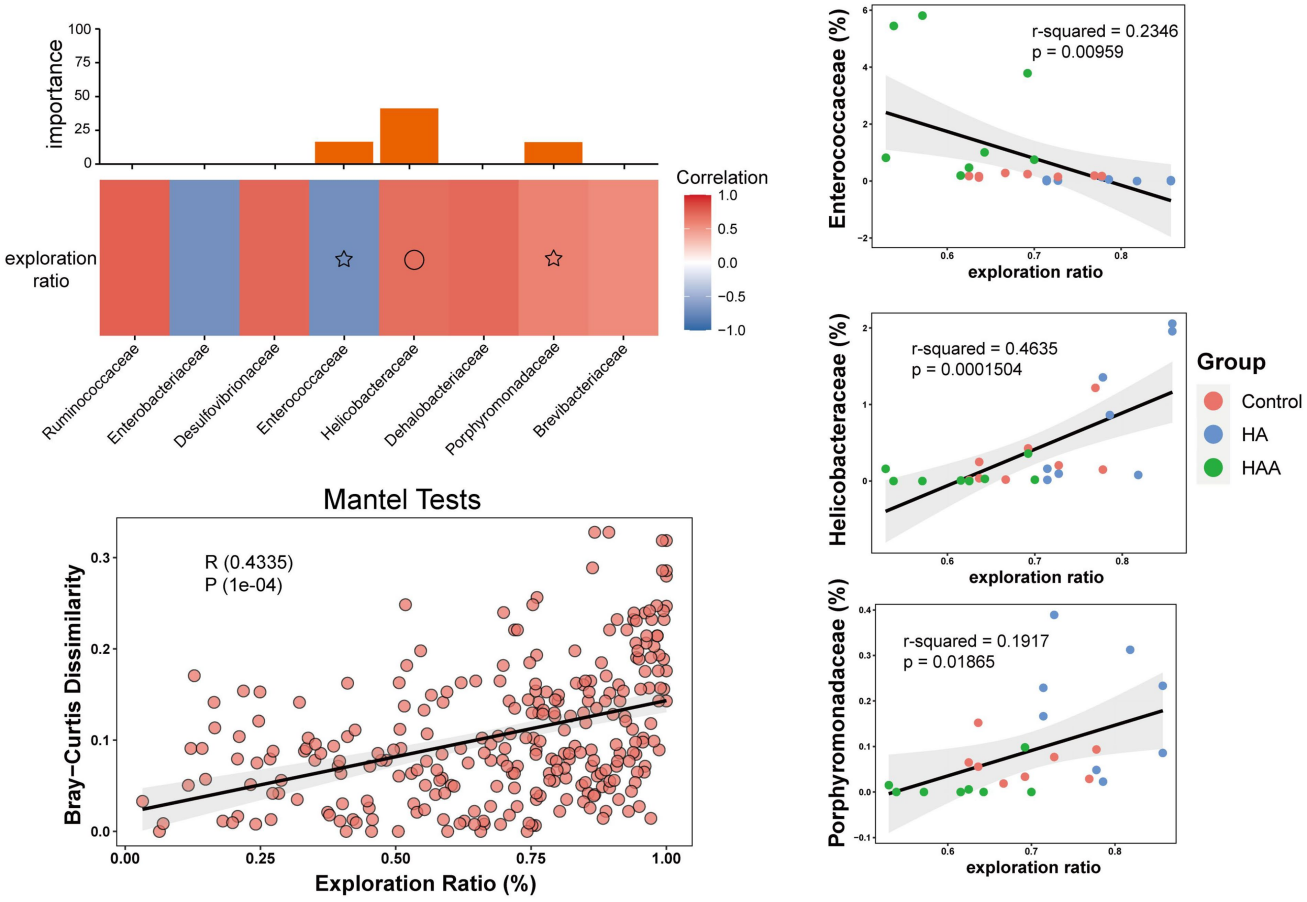
Correlation between microbial community and behavioral test in the colon. **(A)** Different colors represent positive (red) or negative (blue) correlation of important family-level microbes with behavioral results. Circles and asterisks represent different levels of importance. **(B–D)** The different results represent the importance of having three species at the family level in behavioral performance. Red circle: control group. Blue circle: HAE group. Green circle: HAE-A group. **(E)** Mantel Test is a correlation test to determine the correlation between two sets of distance measure matrices. And it is used to evaluate whether the sample distance in one matrix is related to the sample distance in the other matrix.

The results of the Spearman’s correlation analysis and heatmaps showed the degree of association of all highly correlated and significant families with the behavioral results (Figure 9A). Subsequently, the combination with the lowest collinearity was selected using the former selection method to construct a linear model. *Helicobacteraceae*, *Porphyromonadaceae*, and *Enterococcaceae* were identified as microbes with high importance (Figure 9A). Further, we conducted separate modeling for the three families and Mantel test analysis (Figures 9B–E). The results showed that the correlation between *Helicobacteraceae* and the exploration ratio was greater than that of the other two families and even greater than the combined effect of the three families. Therefore, it can be assumed that *Helicobacteraceae* is the most important microbe in the colon that affects the results of behavioral tests.

## Discussion

As an efficient and flexible assay for investigating various aspects of working memory in rodents, the novel object test was applied in this study to assess working memory deficits associated with the prefrontal cortex (Lueptow, 2017). The novel object test relies on the natural predisposition of rodents to explore novelty, and poor working memory ability is reflected by short escape latency and high error (Malekmohamadi et al., 2007; Chen et al., 2014). According to the results of the behavioral tests in this study, high-altitude exposure for 14 days resulted in working memory impairment. The HAE group had a lower exploration rate in the novel object test, indicating lower working memory capacity. This result was consistent with that reported by Ma et al. (2019). Acute exposure to high altitude results in reduced cognitive tasks and working memory, primarily reduced accuracy and responsive behavioral performance, as well as reduced activity in some brain regions, including the medial frontal and visual cortices (Yan et al., 2011). Similar results to acute exposure were found in the long-term exposure group, with subjects who had moved to high altitudes for more than 2 years showing reduced accuracy and slower responses to working memory tasks (Yan et al., 2010). Maintaining the balance of the gut microbiota is considered an effective way to modulate cognitive ability through MGBA (Borre et al., 2014). If the gut microbiota is disturbed, the intestinal environment is affected, which may lead to serious symptoms in the host. For example, damage to the integrity of the intestinal barrier may cause bacteria and/or their metabolites in the lumen to enter blood circulation and eventually impair brain function and cognitive abilities (Julio-Pieper et al., 2014; Sharon et al., 2016). Based on the results of the behavioral test in the present study, MGBA may play an important role during high-altitude exposure, as the mice with antibiotic-disrupted intestinal microbiota performed worse during the novel object test after 14 days of high-altitude exposure. The results of the behavioral test provide new evidence that brain dysfunction caused by high-altitude exposure may be closely related to MGBA.

The P300 results of the oddball behavioral paradigm training test showed the same trend among the three experimental groups in this study. P300 is widely accepted as a marker with positive relativity for all processes of working memory, including formation and retrieval (Ehlers and Somes, 2002). As an event-related potential elicited in response to certain types of stimuli, it is considered a marker of working memory and is closely associated with the formation and retrieval of memories. P300 has also been found to be sensitive to a variety of cognitive processes, including attention, decision-making, and memory encoding, especially in the prefrontal cortex (Eidel and Kübler, 2020). In this study, the P300 evoked by the oddball test in response to rare stimulation significantly decreased in amplitude in the HAE and HAE-A groups, suggesting deficits in working memory. In general, a neuron system may recruit more resources to ‘mark’ an unknown stimulation. By “marking” the stimulation, the neuron system can better process and understand the information, which allows for improved learning and memory formation (Fjell and Walhovd, 2003). Thus, it is more likely that hypoxia may reduce the potential of the brain to recruit more neurons, and this influence of high-altitude exposure is aggravated by disturbed gut microbiota.

The prefrontal cortex is an important brain region for cognition and memory, and growing evidence suggests that prefrontal damage can lead to working memory impairment (Jodo et al., 2004). It is sensitive to pathophysiological changes such as reduced antioxidant capacity, and oxidative damage can impede prefrontal-dependent memory function by reducing the production of new neurons (Huang et al., 2015). A previous study showed that the levels of protein oxidation and catalase activity increased, and serum iron, serum ferritin, and transferrin saturation levels decreased with high-altitude exposure, indicating that the high-altitude environment may affect antioxidant capacity and iron homeostasis (Rytz et al., 2022). The level of antioxidant capacity is closely related to the influence of high altitude, which was also found by Zhang et al. (2015) during research on the effect of hyperbaric oxygen preconditioning in plain migrant plateau personnel. Therefore, we studied the prefrontal cortex to determine the mechanisms by which high-altitude exposure stress induces working memory dysfunction and how intestinal probiotics protect the host from memory dysfunction. CAT, SOD, and GSH-Px are important enzymes that protect against oxidative stress by reducing the activity of superoxide anions and hydrogen peroxide (Thakare et al., 2017). In our study, MDA formation was increased and T-AOC, SOD, GSH-Px, and CAT activities were reduced in the prefrontal cortex of mice exposed to high-altitude stress, which is consistent with the results of Niki (2012). Similar to many other stressors, acute high-altitude exposure impairs neuronal morphology and prefrontal function, and induces dendritic remodeling in the prefrontal cortex, resulting in impaired working memory in humans and animals (Miller et al., 2018). Thus, our findings clearly indicate that oxidative stress was enhanced. This enhancement is at least partially associated with working memory dysfunction induced by high-altitude stress (Freitas et al., 2014; Passiatore et al., 2014).

Oxidative damage triggers apoptosis and impairs learning- and memory-dependent functions (Schafer and Buettner, 2001). It was also found that oxidative stress caused by reactive oxygen species is an important link to apoptosis protein (Miyamoto et al., 2006). In oxidative stress-mediated apoptosis, caspase activation, changes in Bcl-2-related proteins, and cytochrome C release occur repeatedly and significantly impact the apoptotic process (Maity et al., 2012). By detecting the apoptosis level in murine brain tissues, Van Gossum et al. (2017) found increased caspase-3 and Bax protein expression and decreased Bcl-2 protein expression and the Bc1-2/Bax ratio in plateau hypoxic mice. Thus, changes in the levels of oxidative stress and apoptotic proteins provide a good assessment of the corresponding changes in prefrontal working memory function during acute high-altitude exposure. In oxidative stress-mediated apoptosis, caspase activation, changes in Bcl-2-related proteins, and cytochrome C release occur repeatedly and significantly impact the apoptotic process (Maity et al., 2012). Changes in the levels of oxidative stress and apoptotic proteins provide a good assessment of the corresponding changes in the prefrontal working memory function during acute high-altitude exposure. We evaluated the level of apoptosis in the prefrontal cortex by determining apoptosis-related proteins. The process of mitochondria-mediated apoptosis can be briefly described as follows: The leakage of cytochrome C through Bax-formed holes in the mitochondrial membrane triggers the formation of an apoptosome that activates caspase-9; in turn, caspase-9 triggers the activation of caspase-3 and ultimately causes cell apoptosis (Wang et al., 2017). During this process, the increase in Bax and decrease in Bcl-2 levels are related to high vulnerability to apoptotic activation (Freitas et al., 2014). In the present study, acute high-altitude exposure altered the apoptosis-associated proteins in the prefrontal cortex. Simultaneously, antibiotic-disrupted intestinal flora exacerbated the reduction in antioxidant capacity and apoptotic levels in the prefrontal cortex after high-altitude exposure stress. This suggests that the modulation of intestinal flora disruption after high-altitude stress may exert beneficial effects on prefrontal oxidative damage and apoptosis.

Previous studies have found that cognitive dysfunction, especially the decline of working memory, is mediated by the gut microbiota in high-altitude environments, which means that the gut microbial structure is closely related to the hypoxic environment. In this study, PCoA analysis of the colonic microbiota revealed that there were different compartments in the colonic microbiota of mice in the control, HA, and HAA groups. Colonic community diversity was significantly higher in the control group than that in the HA group, and the diversity in the HA group was also significantly higher than that in the HAA group. In addition, the microbial richness in the control group was significantly higher than that in the HA group. These results imply that hypoxic exposure and antibiotic treatment have important effects on colonic microbial communities. The analysis of microbial composition demonstrated the differential abundance of taxa among the different environments. Compared to the control group, the relative abundance of dominant colonic microbes in mice was significantly altered in the HA and HAA groups. To further define the most representative microbes in each group of mice, we used indicator species analysis to identify microorganisms that preferred different environments and subsequently clustered taxonomy with similar evolutionary levels into the same family. We observed *Lactobacillaceae*, which prefers a hypoxic environment, *Lachnospiraceae*, which is sensitive to antibiotics in hypoxic environments, and *S24-7*, which was deficient in the HA and HAA groups compared with the control group. The family *Lactobacillaceae* comprises facultative anaerobic gram-positive rods, belonging to the category of lactic acid bacteria (Suissa et al., 2021). Long-term consumption of *Lactobacillaceae* species induces qualitative and quantitative modifications in the microbial ecosystem of the human gastrointestinal tract (Di Cerbo et al., 2015). Specifically, Lactobacillaceae probiotics possess antitumor, antitoxic, cholesterol-lowering, antibacterial, and antiviral activity (Nazir et al., 2018). Hu et al. (2022) found that the abundance of *Lactobacillaceae* changed with prolonged hypoxic exposure in a hypoxic environment. Based on the above studies, we believe that changes in the abundance of *Lactobacilli* may be a defining feature after hypobaric hypoxia exposure, which is of great significance for future studies. *Lachnospiraceae* are present from early infancy. Although taxa of this family have repeatedly shown their ability to produce beneficial metabolites for the host, the number of *Lachnospiraceae* has also increased in the intestinal lumen of subjects with different diseases (Vacca et al., 2020). Juhász et al. (2021) found that antibiotic treatment led to an increase in the abundance of *Lachnospiraceae* bacteria in the gut of mice, which also indicated that the antibiotic treatment was effective in this study. Alterations in the abundance of the *S24-7* family members are related to different environmental conditions. Previous research has found that *S24-7* is more abundant in diabetes-sensitive mice fed a high-fat diet, particularly when the chow is supplemented with glucose oligosaccharides (Serino et al., 2012) and following treatment-induced remission of colitis in mice (Rooks et al., 2014). Moreover, *S24-7* was also demonstrated to be related to L-valine, leucine, and glutarate, and involved in the metabolism of amino acids, low cholesterol, low-density lipoprotein (LDL), and fatty acid content (Seo et al., 2019). Although these observations are currently limited to murine studies, they suggest that *S24-7* is involved in host–microbe interactions that affect gut function and health.

Finally, to further explore the mechanism of impaired working memory after hypoxic exposure, we analyzed the correlations between all microbial families and behavioral cognitions. The selected important microbes were then modeled with the behavioral results, and the Mantel test was used to establish the combined microbial and behavioral modeling analysis. We found that *Helicobacteraceae*, *Porphyromonadaceae*, and *Enterococcaceae* were highly important microorganisms in terms of their correlation with the behavioral results. Then, through separate modeling and Mantel analysis of the three families, we found that the correlation between *Helicobacteraceae* and exploration rate was greater than that of the other two families, and even higher than the comprehensive effect of the three families. Shen et al. (2017) compared the gut microbiota of APP/PS1 transgenic mice with AD and C57/BL6 wild-type (WT) mice and found that the abundance of *Helicobacteraceae* increased significantly in APP/PS1 mice compared to WT mice. Bäuerl et al. (2018) reported the presence of *Helicobacteraceae* and a higher abundance of *Desulfovibrionaceae* in transgenic mice at the family level. Taken together, it is reasonable to assume that *Helicobacteraceae* are the most likely family of colonic bacteria to influence cognitive dysfunction.

## Conclusion

Disrupted gut microbiota could aggravate working memory dysfunction induced by high-altitude exposure in mice, indicating the existence of a link between high-altitude exposure and MGBA. The most relevant bacterial family, *Helicobacteraceae,* may play an important role in this process.

## Data availability statement

The datasets presented in this study can be found in online repositories. The names of the repository/repositories and accession number(s) can be found at: https://www.ncbi.nlm.nih.gov/, PRJNA884182.

## Ethics statement

All animal experiments were conducted in accordance with the guidelines for the feeding and use of experimental animals (approval no: SYXK2022-023) approved by the Committee of Nanfang Hospital Baiyun Branch, Southern Medical University.

## Author contributions

ZZ, DC, and GW: conceptualization and methodology. DC, HR, and XZ: project administration and data curation. ZZ, WX, and WP: writing—original draft. ZZ, GW, YZ, and LY: writing—review and editing. CL, YH, and HL: supervision. All authors contributed to the article and approved the submitted version.

## Funding

This work is supported by the Scientific Development funds for Local Region from the Chinese Government in 2022 (XZ202201YD0018C), Cultivation Fund of National Nature Science Foundation (grant no. qiankehe2018-5764-11), and Natural Science Foundation of Guangdong Province, China (grant no. 2022A1515011230).

## Conflict of interest

The authors declare that the research was conducted in the absence of any commercial or financial relationships that could be construed as a potential conflict of interest.

## Publisher’s note

All claims expressed in this article are solely those of the authors and do not necessarily represent those of their affiliated organizations, or those of the publisher, the editors and the reviewers. Any product that may be evaluated in this article, or claim that may be made by its manufacturer, is not guaranteed or endorsed by the publisher.

## Footnotes

^1^ https://docs.qiime2.org/
